# Cell-Free DNA Versus Circulating Tumor Cells: A Pilot Study of Alpha-Fetoprotein Analysis for Diagnosis and Treatment Monitoring in Hepatocellular Carcinoma

**DOI:** 10.3390/bios15090579

**Published:** 2025-09-04

**Authors:** Ga Young Moon, Hyun Sung Park, Ha Neul Kim, Hei-Gwon Choi, Yonghan Han, Hyuk Soo Eun, Tae Hee Lee, Jiyoon Bu

**Affiliations:** 1 Department of Biological Sciences and Bioengineering, Inha University, 100 Inha-ro, Michuhol-gu, Incheon 22212, Republic of Korea; gayeong056@inha.edu (G.Y.M.); hyunseong@inha.edu (H.S.P.); choko9219@inha.edu (Y.H.); 2 Department of Medical Science, Chungnam National University, 266 Munhwa-ro, Jung-gu, Daejeon 35015, Republic of Korea; tsb04254@o.cnu.ac.kr (H.N.K.); hundred4120@cnu.ac.kr (H.-G.C.); 3 Department of Internal Medicine, College of Medicine, Chungnam National University, 266 Munhwa-ro, Jung-gu, Daejeon 35015, Republic of Korea; 4 Department of Internal Medicine, Chungnam National University Hospital, 282 Munwha-ro, Jung-gu, Daejeon 35015, Republic of Korea; 5 Department of Biomedical Laboratory Science, Daegu Health College, Chang-Ui Building, 15 Yeongsong-ro, Buk-gu, Daegu 41453, Republic of Korea; 6 Biomedical Research Institute, Inha University Hospital, 27 Inhang-ro, Jung-gu, Incheon 22332, Republic of Korea; 7 Department of Biological Engineering, Inha University, 100 Inha-ro, Michuhol-gu, Incheon 22212, Republic of Korea; 8 Biohybrid Systems Research Center, Inha University, 100 Inha-ro, Michuhol-gu, Incheon 22212, Republic of Korea

**Keywords:** circulating tumor cells (CTCs), cell-free DNA (cfDNA), alpha-fetoprotein (AFP), hepatocellular carcinoma (HCC), multimodal liquid biopsy

## Abstract

Serum alpha-fetoprotein (AFP) is widely used for hepatocellular carcinoma (HCC) management, yet its limited sensitivity and specificity restrict diagnostic and prognostic utility. In this study, we explore the clinical potential of AFP quantification from cell-free DNA (cfDNA) and circulating tumor cells (CTCs) using a novel bead-based liquid biopsy platform. Following isolation, AFP abundance in cfDNA was quantified by qPCR, while AFP protein expression in CTCs was assessed via immunohistochemistry. Compared to serum AFP, cfDNA-derived AFP demonstrated significantly greater diagnostic accuracy in distinguishing HCC patients from non-cancerous individuals (*p* < 0.0001, AUC = 0.998), while AFP^+^ CTCs showed high specificity. Post-treatment changes in AFP levels from cfDNA and CTCs were significantly associated with therapeutic response and overall survival, outperforming conventional serum AFP. Longitudinal monitoring further revealed that cfDNA AFP levels reliably captured recurrence events prior to clinical diagnosis. Moreover, a combined metric integrating AFP levels from cfDNA and CTCs significantly improved response stratification (AUC = 0.89), outperforming individual biomarkers. This pilot study highlights the potential of multimodal AFP profiling through cfDNA and CTCs as a promising, non-invasive approach for enhancing diagnosis, prognosis, and treatment monitoring in HCC, with direct implications for personalized therapeutic strategies.

## 1. Introduction

Despite advances in clinical diagnostics, hepatocellular carcinoma (HCC) continues to elude early detection, driving its position as one of the leading causes of cancer-related death worldwide [1,2]. The current diagnostic gold standard integrates imaging modalities—such as computed tomography (CT) and magnetic resonance imaging (MRI)—with serum biomarker quantification, particularly alpha-fetoprotein (AFP) [3]. AFP is a biologically actionable molecule in HCC, as it has been implicated in tumor growth, angiogenesis, and immune modulation [4]. Specifically, AFP has been shown to influence cellular pathways involved in proliferation and immune evasion, highlighting its role in HCC progression [5]. Owing to its affordability and accessibility, serum AFP quantification remains widely used in clinical practice. However, merely quantifying serum AFP provides limited sensitivity and specificity, which significantly restricts its utility, especially in early-stage HCC. For instance, a retrospective study reported that an AFP cutoff of >20 ng/mL achieved a sensitivity of 70.1% and specificity of 89.8% for overall HCC detection [6]. In contrast, for early-stage HCC, sensitivity dropped to 49.3%, with an area under the receiver operating characteristic curve (AUC-ROC) of 0.62, indicating poor discriminative performance. Moreover, AFP elevations are not specific to malignancy, as they are frequently observed in benign liver conditions such as cirrhosis and chronic hepatitis. Notably, one study reported that among patients with AFP levels > 20 ng/mL but no detectable liver tumors on ultrasound, the specificity for HCC diagnosis was only 44% [7].

Due to the limited sensitivity and specificity of serum AFP, there is increasing interest in identifying more reliable liquid biopsy biomarkers for the diagnosis and prognostic evaluation of HCC [8]. Specifically, circulating tumor DNA (ctDNA) and circulating tumor cells (CTCs) have attracted considerable attention as potential candidates. ctDNA is released into the bloodstream through apoptosis or necrosis of tumor cells and represents a tumor-derived subset of cell-free DNA (cfDNA) [9,10]. Patients with cancer typically exhibit elevated cfDNA levels due to increased cell death and disintegration associated with uncontrolled tumor growth [11]. Since ctDNA carries tumor-specific genetic and epigenetic alterations, it can be leveraged for early detection, molecular profiling, and disease monitoring [12,13]. Meanwhile, CTCs are viable tumor cells shed from primary or metastatic lesions into the circulation, where they can initiate distant metastases [14]. Although extremely rare in circulation, these tumor cells provide both genetic and phenotypic information related to tumor burden, enabling characterization of tumor heterogeneity, metastatic potential, and therapeutic response at the cellular level.

Recent efforts have explored AFP-based analyses of cfDNA and CTCs to improve the diagnostic accuracy of HCC [15,16]. While direct analysis of AFP gene expression in cfDNA is not feasible, quantification of AFP genomic DNA fragments has been proposed as an alternative approach for identifying the presence of HCC. For example, Kim et al. quantified AFP gene abundance in plasma cfDNA using qPCR normalized to a reference gene, and demonstrated that this approach successfully distinguished HCC patients from healthy donors and individuals with benign liver disease [17]. The study reported high diagnostic accuracy, with AUC-ROC values of 0.861, 0.744, and 0.971 when differentiating HCC from cirrhosis, alcoholic hepatitis, and healthy individuals, respectively. Similarly, the analysis of AFP protein expression in CTCs has demonstrated diagnostic relevance. A study utilizing a cell block method for multiple immunohistochemical analyses confirmed that AFP protein could be detected in CTCs from HCC patients, supporting their potential role as diagnostic and prognostic biomarkers [18]. These approaches may improve the sensitivity and specificity of HCC detection, particularly in cases where serum AFP levels are inconclusive.

In this study, we investigated whether AFP measurements derived from cfDNA and CTCs could enhance the diagnostic and prognostic performance for HCC compared to conventional serum AFP testing. Our group previously developed an alginate bead-based liquid biopsy platform capable of capturing cfDNA from plasma using polydopamine–silica hybrid (PDA/SiO_2_)-coated beads and isolating CTCs from the peripheral blood mononuclear cell (PBMC) layer using anti-EpCAM (aEpCAM)-functionalized beads [13]. Using these systems, we quantified AFP gene abundance in plasma cfDNA by qPCR normalized to a reference gene, and evaluated intracellular AFP protein expression in CTCs by immunohistochemistry following a protocol analogous to cell block-based analysis. These cfDNA- and CTC-derived AFP measurements were compared with serum AFP levels routinely measured in clinical practice, as well as liver function markers including aspartate aminotransferase (AST), alanine aminotransferase (ALT), alkaline phosphatase (ALP), and albumin. We systematically assessed the diagnostic accuracy and prognostic value of AFP across these sources and further explored whether combining cfDNA- and CTC-based AFP measurements could provide synergy in prognostic utility beyond individual markers. Through comprehensive assessment of AFP levels across cfDNA and CTCs, this study establishes a multimodal liquid biopsy approach that enhances the diagnostic precision and real-time monitoring of HCC, offering a clinically translatable strategy for advancing personalized oncology.

## 2. Materials and Methods

### 2.1. Patient Recruitment and Sample Collection

Blood samples from patients diagnosed with HCC were prospectively collected at Chungnam National University Hospital. Blood samples from non-cancerous individuals were obtained from healthy volunteer donors. All procedures involving human participants were conducted in accordance with institutional ethical guidelines and approved by the Institutional Review Board of Chungnam National University Hospital (IRB No. 2020-10-088-025).Written informed consent was obtained from all participants prior to sample collection. All research was conducted in accordance with both the Declarations of Helsinki and Istanbul.

### 2.2. Assessment of Therapeutic Response in HCC Patients

Tumor response was primarily assessed by radiological imaging using contrast-enhanced CT or MRI. Pre- and post-treatment images were compared according to the Modified Response Evaluation Criteria in Solid Tumors (mRECIST v1.1), which evaluates the viable tumor burden based on areas of arterial phase enhancement. In parallel, serum levels of AFP, AST, ALT, and albumin were measured before and after treatment. Changes in these biomarkers were analyzed relative to baseline to support radiological assessment. Note that the evaluation of the diagnostic and prognostic performance of these markers is consistent with current clinical guidelines.

### 2.3. Bead Fabrication for the Capture of CTCs and cfDNA

Alginate beads were fabricated as described in our previous study [13]. Briefly, a 5% (*w*/*v*) sodium alginate solution was added dropwise into deionized water containing 100 mM calcium chloride using a 200 µL pipette under gentle stirring at room temperature for 1 h. The cross-linked alginate beads were collected, washed with deionized water, and stored at 4 °C until further use.

For CTC isolation, beads were functionalized with anti-EpCAM antibodies (1:100 dilution, eBioscience; clone 1B7 in PBS) via EDC/NHS coupling. Beads were first activated by incubation in 0.5 M MES buffer (pH 6.0) containing 200 mM 1-ethyl-3-(3-dimethylaminopropyl) carbodiimide hydrochloride (EDC) and 100 mM N-hydroxysuccinimide (NHS) for 10 min at room temperature. Subsequently, beads were incubated with anti-EpCAM antibodies at 4 °C for 12 h. Unbound antibodies were removed by washing with PBS.

For cfDNA capture, beads were modified with polydopamine–silica. Alginate beads were first incubated with 5 mM dopamine hydrochloride for 12 h under mildly alkaline conditions (adjusted to pH~7.0 using Tris–HCl buffer). After washing with deionized water, the beads were incubated in 1 mL of silica solution for 1 h to complete the PDA/SiO_2_ coating process prior to use in DNA analysis.

### 2.4. Scanning Electron Microscopy and Energy Dispersive X-Ray Spectrometry Analysis

Surface morphology and elemental composition of the beads were characterized by SEM/EDS (S-4300SE, Hitachi, Tokyo, Japan). Samples were sputter-coated with platinum (Pt) at 20 mA for 120 s (tooling factor 2.30). SEM imaging was performed at an accelerating voltage of 5–15 kV with a working distance of 13.4–14.0 mm; EDS spectra were collected at 5–15 kV.

### 2.5. Assessment of aEpCAM-Bead Capture Efficiency

The cancer cell capture capability of aEpCAM-coated alginate beads was evaluated using the Hep3B hepatocellular carcinoma (HCC) cell line. Hep3B cells were cultured in Dulbecco’s Modified Eagle Medium (DMEM) supplemented with 10% fetal bovine serum (FBS) and 1% penicillin–streptomycin (P/S). Prior to the capture assay, cells were stained with DiI (Invitrogen, Carlsbad, CA, USA; V22885) and seeded into 96-well plates at a density of 5000 cells per well in 100 μL of medium. A single aEpCAM-coated bead was then introduced into each well and incubated with the cells for 1 h at room temperature. Following incubation, residual unbound cells were imaged using fluorescence microscopy, and capture efficiency was quantified with ImageJ software.

### 2.6. Confirmation of Antibody Immobilization

Antibody conjugation was verified by incubating aEpCAM-coated beads with Alexa Fluor 488–labeled secondary antibody for 1 h at room temperature. Following incubation, the beads were gently washed with PBS, and fluorescence signals were visualized by fluorescence microscopy in comparison with bare alginate beads. In parallel, a plate reader was used to quantify the fluorescence intensity of the residual secondary antibody solution.

### 2.7. cfDNA Isolation Using Bead-Based Assay

Plasma was collected from 3–5 mL of human whole blood after centrifugation at 2990 rpm for 10 min. The plasma was pre-treated with proteinase K at a 10:1 volume ratio (200 µL:20 µL), followed by the addition of 200 µL of lysis buffer and incubation for 30 min at 37 °C. Subsequently, 200 µL of 95% ethanol was added to the mixture. PDA/SiO_2_-coated beads were immersed in the pre-treated samples for 10 min under gentle agitation. After incubation, the beads were washed with 350 µL of AW1 buffer (Qiagen, Hilden, Germany) and stored in 50 µL of RNase/DNase-free water.

### 2.8. qPCR Analysis of AFP Levels in cfDNA

qPCR was performed using the CFX Opus 96 Real-Time PCR System (Bio-Rad, Hercules, CA, USA) and the 2× QuantiNova SYBR Green Master Mix (Qiagen, Germany), according to the manufacturer’s protocol. Each 20 µL reaction contained 10 µL of 2× SYBR Green Master Mix, 1.1 µL of plasma-derived cfDNA, 1.4 µL of each primer (10 µM), and 6.1 µL of nuclease-free water. The thermal cycling conditions were as follows: initial denaturation at 95 °C for 2 min, followed by 40 cycles of 95 °C for 5 s and 60 °C for 10 s. All reactions were performed in quadruplicate. The expression of AFP was normalized to the reference gene GAPDH using the 2^−∆Ct^. Primer sequences were as follows: an AFP forward primer (5′- GCA GAG GAG ATG TGC TGG ATT G-3′), AFP reverse primer (5′-CGT GGT CAG TTT GCA GCA TTC TG-3′), GAPDH forward primer (5′-GGG TCT TTG CAG TCG TAT GG-3′), and GAPDH reverse primer (5′-CCC CAG CTA CAG AAA GGT CA-3′).

### 2.9. CTC Isolation Using Bead-Based Assay

Whole blood samples were processed by density gradient centrifugation of PBS-diluted blood (1:1) layered over Ficoll-Paque Plus (GE Healthcare, Chicago, IL, USA). The peripheral blood mononuclear cell (PBMC) layer was carefully collected and washed once with PBS. aEpCAM-functionalized beads were then incubated with the PBMC layer for 1 h under gentle agitation to facilitate CTC capture. Following incubation, the beads were washed with PBS to remove unbound cells. Captured cells were released by incubating the beads in PBS containing 0.5 M EDTA (pH 8.0) for 10 min. The released cells were collected by centrifugation at 1000× *g* for 3 min, and the supernatant was discarded. To attach the cells to slides for downstream analysis, 100 µL of the cell pellet was deposited onto a microscope slide and dried on a hot plate at 60 °C.

### 2.10. AFP Immunohistochemistry Analysis of CTCs

The slides were immediately fixed with 4% paraformaldehyde for 15 min, followed by antigen retrieval using a pre-heated buffer solution containing 10 mM sodium citrate and 0.05% Tween 20 (pH 6.0) at 90 °C for 60 min. After cooling at room temperature for 10 min, endogenous peroxidase activity was quenched, and the slides were blocked with 1% bovine serum albumin (BSA) to prevent nonspecific antibody binding. The slides were then incubated with a primary antibody against AFP (clone 1E8, eBioscience, Diego, CA, USA; 1:100 dilution in PBS) for 1 h, followed by three washes with DAKO wash buffer. Detection was performed using the DakoREAL EnVision detection system (Dako, Glostrup, Denmark) for 40 min. After another three washes, the slides were incubated with 3,3′-diaminobenzidine tetrahydrochloride (DAB) (DAKO) for 10 min and counterstained with Mayer’s hematoxylin. AFP-positive CTCs were identified based on the following criteria: (1) unequivocal brown cytoplasmic staining; (2) cell diameter greater than 12 µm; and (3) a high nuclear-to-cytoplasmic ratio relative to background leukocytes.

### 2.11. Statistics

All statistical analyses were performed using GraphPad Prism (v10.5.0; Dotmatics, Boston, MA, USA) and SPSS (v29.0.2.0; IBM Corp., Armonk, NY, USA). Group comparisons for continuous variables were conducted using the Mann–Whitney *U* test or Kruskal–Wallis test for nonparametric data, and Student’s *t*-test for normally distributed variables. Categorical variables were analyzed using the chi-square test or Fisher’s exact test.

AUC-ROC curves were generated to evaluate diagnostic performance, with the area under the curve calculated along with 95% confidence intervals (CI). Differences between AUCs and the null hypothesis (AUC = 0.5) were assessed using the DeLong test. Survival outcomes were analyzed using Kaplan–Meier estimates and compared via the log-rank test. A two-sided *t*-test was performed to analyze the statistical significance.

### 2.12. Combined AFP Level Calculation

The combined AFP level was used to evaluate patients’ responses to treatment and was calculated by summing two parameters: (1) the change in relative AFP abundance in cfDNA (log2^−ΔCt^) between pre- and post-treatment samples, and (2) the post-treatment AFP^+^ CTC counts (cells/mL). These two parameters were directly added to generate the combined AFP level, which was then used to classify patients into response categories of complete response (CR), partial response (PR), stable disease (SD), or progressive disease (PD).

## 3. Results

### 3.1. Measurement of AFP in Liquid Biopsy Biomarkers

**Figure 1:**
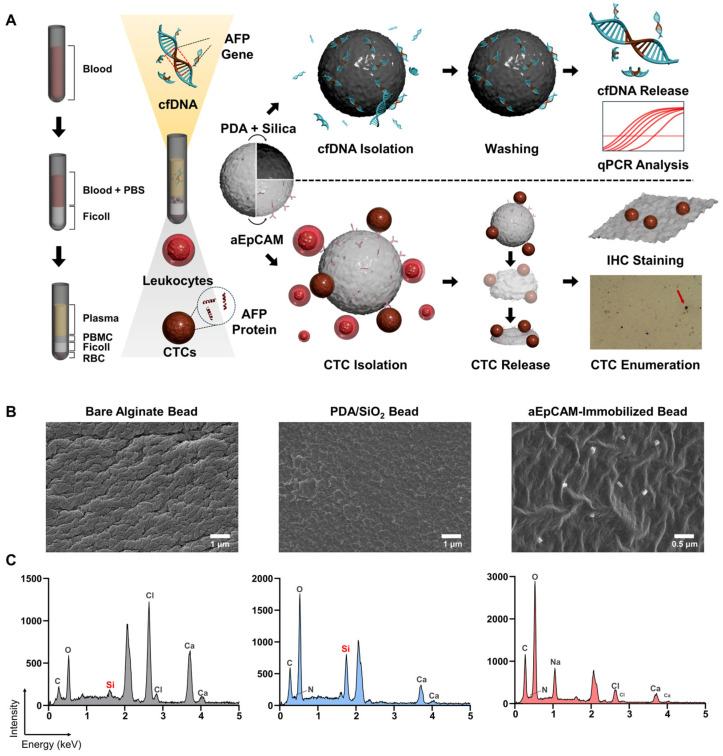
(**A**) Schematic overview of AFP quantification from cfDNA and CTCs using a bead-based liquid biopsy platform for the diagnosis and prognosis of HCC. cfDNA was captured from serum using PDA/SiO_2_-coated alginate beads and AFP abundance was quantified by qPCR. CTCs were isolated from the PBMC layer using aEpCAM-functionalized beads, released, and analyzed by IHC for AFP expression. AFP levels from cfDNA and CTCs were compared with serum AFP to evaluate their diagnostic and prognostic relevance in HCC. (**B**,**C**) Verification of surface functionalization of PDA/SiO_2_- and aEpCAM-coated alginate beads using SEM–EDS.

Fifty-seven subjects were enrolled in this study, including 47 patients with HCC and 10 without malignant disease (Table S1). Blood samples were processed by density gradient centrifugation using Ficoll-Paque to separate the PBMC layer and serum, according to the manufacturer’s instruction. AFP levels were measured across different liquid biopsy biomarkers, including serum proteins, cfDNA, and CTCs (Figure 1A). Briefly, serum AFP concentrations were quantified using an automated electrochemiluminescence immunoassay (ECLIA) immediately following serum separation. In parallel, cfDNA and CTCs were isolated from blood samples using alginate beads functionalized with PDA/SiO_2_ and aEpCAM, respectively, as described in our previous study (Figure S1); cfDNA was adsorbed onto PDA/SiO_2_-coated beads by incubation with serum, whereas CTCs were captured on aEpCAM-functionalized beads from the PBMC layer [13]. Upon isolation of cfDNA, AFP gene abundance was quantified by qPCR, with normalization to the reference gene (GAPDH) using the 2^–ΔCt^ method. For CTCs, cells captured on aEpCAM-coated beads were released by bead dissolution and subjected to IHC staining with anti-AFP antibodies. Cells exhibiting unequivocal brownish cytoplasmic staining for AFP, measuring larger than 10 µm, and displaying a higher nuclear-to-cytoplasmic (N/C) ratio compared to background leukocytes were identified as AFP^+^ CTCs (Figure S2). Further methodological details are provided in the Experimental Section, along with the decision tree illustrated in Figure S3.

Note that prior to the treatment of clinical samples, surface functionalization of PDA/SiO- and aEpCAM-coated beads was validated using SEM–EDS, as indicated by the strong Si signal from PDA/SiO_2_-coated beads and the presence of aEpCAM confirmed by SEM imaging of aEpCAM-coated beads (Figure 1B,C). In addition, incubation with Alexa Fluor 488–labeled secondary antibodies further verified the presence of aEpCAM on the alginate bead surface (Figure S4). Target-specific capture capability was also demonstrated by incubating PDA/SiO_2_-coated beads with fluorescently labeled nucleic acids and aEpCAM-coated beads with Hep3B HCC cells. PDA/SiO_2_-coated beads exhibited a 2.83-fold stronger fluorescent signal compared to unmodified beads (*p* = 0.001), while the capture efficiency of Hep3B cells on aEpCAM-coated beads was 7.14 ± 1.74%, consistent with our previous findings (Figures S5 and S6) [11,12,13].

### 3.2. Diagnostic Utility of AFP Derived from Liquid Biopsy Biomarkers

**Figure 2:**
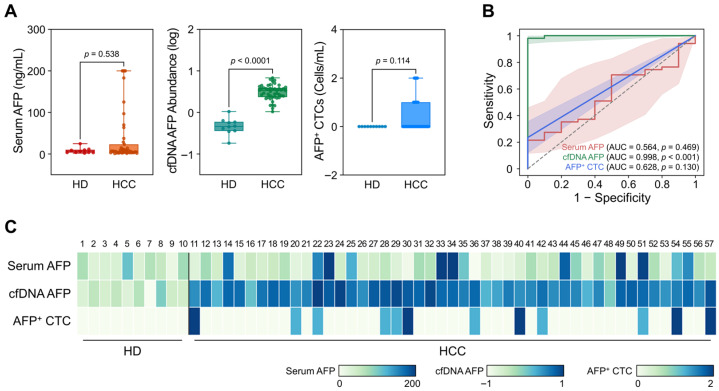
Diagnostic capability of AFP measured from serum, cfDNA, and CTCs in HCC patients. (**A**) Box plots comparing AFP levels across serum (ng/mL), cfDNA (log_10_[2^–ΔCt^]), and CTCs (cells/mL) between HCC patients and non-cancerous individuals. Serum AFP levels showed no significant difference (*p* = 0.538), while cfDNA-derived AFP abundance was significantly elevated in HCC patients (*p* < 0.0001). AFP^+^ CTC counts were not significantly different (*p* = 0.114), though they were detected only in HCC samples. (**B**) ROC analysis comparing the diagnostic performance of serum AFP (AUC = 0.564), cfDNA-derived AFP (AUC = 0.998), and AFP^+^ CTCs (AUC = 0.628). (**C**) Heatmap of AFP levels measured from serum, cfDNA, and CTCs across all participants.

As shown in Figure 2A, serum AFP protein levels did not effectively differentiate HCC patients from non-cancerous individuals. The median serum AFP concentration was higher in HCC patients (6.73  ±  3.82 ng/mL) compared to non-cancerous individuals (5.07  ±  2.53 ng/mL); however, this difference did not reach statistical significance (*p* = 0.538). Note that patients with serum AFP concentrations greater than 200 ng/mL (*n* = 4) were omitted from graphical representation, presented as 200 ng/mL, but remained included in the overall statistical analysis. In contrast, AFP gene abundance in cfDNA showed a significant difference between HCC patients and non-cancerous individuals. Specifically, the median log_10_(2^–ΔCt^) value, representing the relative abundance of AFP normalized to GAPDH, was 0.53  ±  0.12 for HCC patients and −0.34  ±  0.10 for non-cancerous individuals (*p* < 0.0001). Meanwhile, AFP^+^ CTC counts did not demonstrate a statistically significant difference between the two groups (*p* = 0.114). However, it should be noted that no AFP^+^ CTCs were detected in non-cancerous individuals, whereas HCC patient samples yielded a mean of 0.36  ±  0.67 CTCs/mL, highlighting the high specificity of AFP^+^ CTCs.

ROC analyses were subsequently conducted to assess the diagnostic performance of each biomarker (Figure 2B). AUC-ROC was measured as 0.564 (95% CI: 0.391–0.737; *p* = 0.469), 0.998 (95% CI:0.991–1.000; *p* < 0.001), and 0.628 (95% CI: 0.462–0.793; *p* = 0.130) for serum AFP, cfDNA, and CTCs, respectively. Notably, the maximum Youden index (J) of AFP gene abundance in cfDNA (J = 0.98 at a threshold of 0.059) outperformed conventional serum AFP (J = 0.23 at a threshold of 26.7 ng/mL) in distinguishing HCC patients from non-cancerous individuals (Figure S7). Meanwhile, AFP^+^ CTCs demonstrated high specificity for HCC diagnosis, but due to the inherent limitation of rarity, the Youden index achieved by AFP^+^ CTCs was only 0.26 at a threshold of ≥1 cell/mL. A heatmap representing AFP levels from each biomarker for individual participants (Figure 2C) further supported the strong clinical utility of cfDNA-derived AFP gene abundance and the high specificity of AFP^+^ CTCs for HCC diagnosis.

The expression levels of additional liver function markers, including AST, ALT, ALP, and albumin, were also evaluated in serum. Although ALP, AST, and albumin exhibited significant differences between HCC patients and non-cancerous individuals, their diagnostic performance, as reflected by AUC-ROC and maximum Youden index values, was inferior to that of AFP gene abundance measured from cfDNA (Figures S7–S9). These findings demonstrate that AFP gene abundance measure from cfDNA offers superior diagnostic capabilities compared to conventional serum AFP and other liver function markers in serum, demonstrating its potential as a more reliable biomarker for HCC detection.

### 3.3. Changes in AFP Levels and Association with Treatment Response

Blood samples were further collected from patients following the first treatment cycle with various modalities, determined based on individualized clinical judgment, which include radiofrequency ablation (RFA), trans-arterial chemoembolization (TACE), surgical resection, and/or radiotherapy (Table S2). Among the 42 HCC patients, 23 demonstrated tumor size reduction following treatment, corresponding to responders who achieved complete responses (CR) or partial responses (PR). In contrast, the remaining 19 patients exhibited stable disease (SD) or progressive disease (PD), and were classified as non-responders.

**Figure 3:**
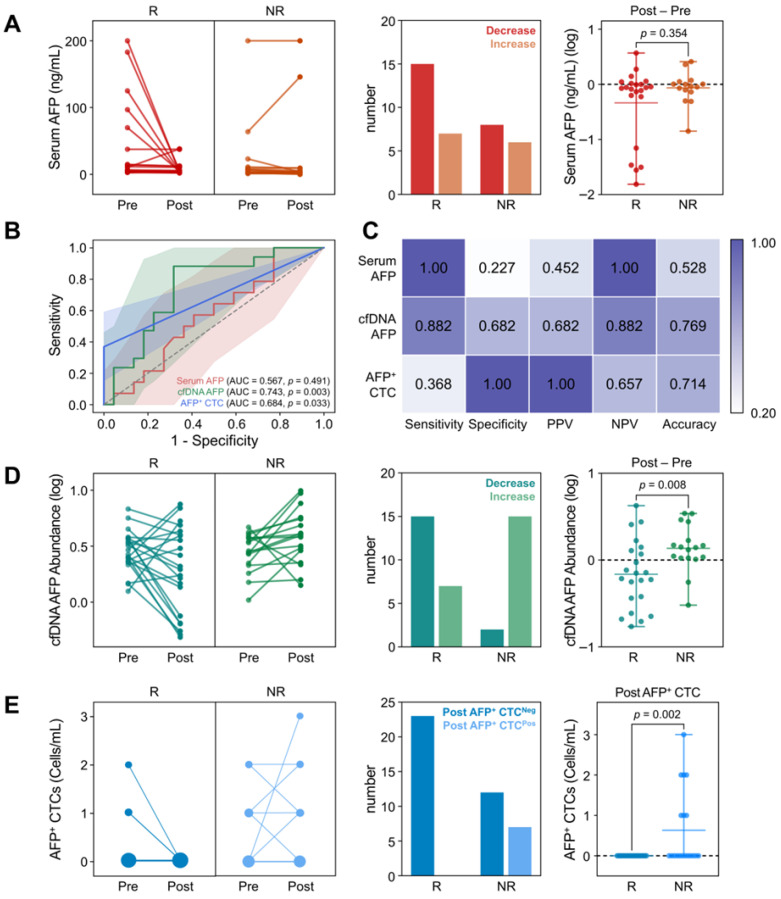
Changes in AFP levels from serum, cfDNA, and CTCs, following the first treatment cycle. (**A**) Changes in serum AFP levels between responders (CR/PR) and non-responders (SD/PD), upon treatment (**B**) ROC curves comparing the predictive performance of AFP changes across serum (AUC = 0.567), cfDNA (AUC = 0.743), and CTCs (AUC = 0.684). (**C**) Comparison of diagnostic capability at Youden index for each biomarker. (**D**) Changes in AFP levels in cfDNA and (**E**) the number of AFP^+^ CTC counts between responders and non-responders upon treatment.

As demonstrated in Figure 3A and Table S3, changes in serum AFP levels did not correlate with treatment response. Although 68.2% of responders exhibited a decrease in serum AFP levels with a median log change in −0.07  ±  0.11, a decrease was also frequently observed in non-responders, with a median log change in −0.04  ±  0.07 (*p* = 0.354). Consequently, ROC analysis demonstrated that changes in serum AFP levels yielded an AUC-ROC of only 0.567 (95% CI: 0.377–0.756; *p* = 0.491) for determining treatment response (Figure 3B). Similarly, the maximum Youden index was only 0.23, further revealing the poor predictive capability of serum AFP levels for determining responders (Figure 3C).

In contrast, changes in AFP abundance in cfDNA were indicative of patient responses. As shown in Figure 3D, 88.2% of patients who exhibited a decrease in cfDNA AFP abundance were identified as responders. Specifically, 68.2% of responders demonstrated a decrease in AFP cfDNA abundance after treatment, with a median log change in −0.18 ± 0.27. This was significantly lower than that observed in non-responders (0.14  ±  0.12; *p* = 0.008), among whom 88.2% exhibited an increase in AFP abundance following treatment. ROC analysis and Youden index measurements further supported this finding, with an AUC-ROC of 0.743 (95% CI: 0.585–0.902; *p* = 0.003) and a maximum Youden index of 0.56 at a threshold log change in −0.0093 (Figure 3B,C).

For AFP^+^ CTCs, dynamic changes in CTC counts were insufficient to distinguish responders from non-responders due to their overall low abundance (Figure 3E). However, notably, all patients who exhibited AFP^+^ CTCs after treatment were classified as non-responders. The average AFP^+^ CTC count after treatment was 0.63  ±  0.96 cells/mL (*p* = 0.002) in non-responders, with a CTC positivity rate of 36.8% (7/19), whereas no AFP^+^ CTCs were detected among responders. Consequently, ROC analysis demonstrated that AFP^+^ CTC counts in follow-up samples could distinguish non-responders from responders with an AUC-ROC of 0.684 (95% CI: 0.515–0.853; *p* = 0.033) and a Youden index of 0.37 at a threshold of ≥1 AFP^+^ CTC/mL. Although the overall performance was lower than that of cfDNA, the high specificity of AFP^+^ CTCs highlights their potential utility as an adjunctive marker for the identification of non-responders.

It should also be noted that the dynamic changes in expression levels of other liver function markers, including AST, ALT, ALP, and albumin, did not show clinical relevance with patients’ therapeutic outcomes. Specifically, ROC analysis demonstrated that these serum biomarkers exhibited significantly lower AUC-ROC values than AFP abundance in cfDNA (Figure S10) and did not show statistical significance in predicting patient responses (*p* > 0.1). Collectively, these findings suggest that while changes in serum protein markers have limited predictive capabilities, cfDNA-based AFP quantification offers superior accuracy for monitoring treatment response, and AFP^+^ CTC detection provides high specificity for identifying non-responders, supporting their complementary roles in improving response assessment in HCC.

### 3.4. Prediction and Monitoring of Patients Survival Based on Changes in AFP Levels

**Figure 4:**
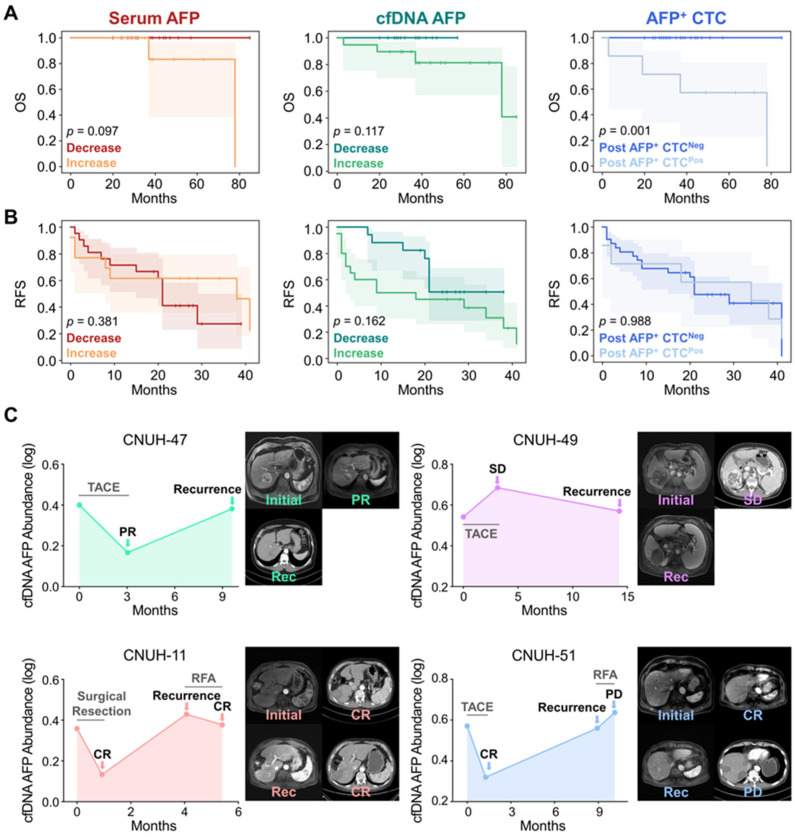
Prognostic significance and recurrence monitoring based on AFP dynamics in serum, cfDNA, and CTCs. (**A**) KM curves showing OS stratified by changes in AFP levels following the first treatment cycle. Decreased AFP levels in serum, cfDNA, and CTCs were associated with improved survival. (**B**) KM curves for RFS indicate no statistically significant differences across the biomarkers. (**C**) Longitudinal monitoring of AFP abundance in cfDNA for two patients (CNUH-47 and CNUH-49) who developed recurrence after initial treatment (upper two panels). Extended longitudinal profiling of cfDNA-derived AFP levels in patients CNUH-11 and CNUH-51, who experienced recurrence and received second-line therapy (bottom two panels).

Kaplan–Meier (KM) survival analysis was performed to assess the prognostic significance of AFP levels derived from serum, cfDNA, and CTCs following the first treatment cycle. Although only four deaths were recorded during the observation period, KM analysis demonstrated that all patients with decreased AFP levels in either serum or cfDNA, as well as those without detectable AFP^+^ CTCs post-treatment, remained alive throughout follow-up (Figure 4A). Notably, post-treatment AFP^+^ CTC counts showed the strongest association with overall survival (OS; *p* = 0.001), with 57.1% (4/7) of patients harboring detectable AFP^+^ CTCs after therapy succumbing during the follow-up period, indicating a statistically significant correlation with adverse survival outcomes.

In contrast, changes in AFP levels following the first treatment cycle were not strongly associated with tumor recurrence-free survival (RFS) (Figure 4B). Notably, patients with increased serum AFP levels exhibited longer RFS of 26.2 months (95% CI: 15.7–36.8 months) compared to those with decreased serum AFP levels (21.9 months; 95% CI: 15.9–28.0 months; *p* = 0.381), a trend contrary to conventional expectations. Meanwhile, patients with increased cfDNA-derived AFP levels following the first treatment cycle tended to exhibit shorter RFS (19.4 months; 95% CI: 11.6–27.2 months) compared to those with decreased levels (27.6 months; 95% CI: 22.2–33.0 months; *p* = 0.162) but the results were statistically insignificant. For CTCs, there was also no significant difference in RFS between patients with (24.9 months; 95% CI:11.3–38.5 months) and without detectable AFP^+^ CTCs post-treatment (24.1 months; 95% CI: 18.3–30.0 months; *p* = 0.988).

The absence of a significant association between changes in AFP levels and tumor recurrence may be explained by the temporal gap between follow-up sample collection and the actual onset of recurrence. In our study, follow-up samples were collected within 3 months after the first treatment cycle, a time frame that likely captured only the immediate treatment effect rather than long-term tumor dynamics. By contrast, the average time to recurrence was 9.2 months, substantially longer than the sampling interval, suggesting that early AFP changes may not fully reflect later recurrence events that develop over an extended period. To further investigate this, we longitudinally monitored four patients who developed recurrence and measured AFP levels across different liquid biopsy biomarkers over time. Interestingly, longitudinal changes in cfDNA-derived AFP levels demonstrated a strong correlation with recurrence dynamics. Patient CNUH-47, who initially achieved PR following TACE treatment, developed recurrence at 9.6 months. At the time of recurrence, an increase in AFP abundance in cfDNA was observed (Figure 4C). In contrast, patient CNUH-49, who exhibited SD after the first TACE cycle, showed an increase in AFP levels during the initial treatment phase. Although AFP abundance decreased at the time of recurrence, it remained elevated compared to the baseline level.

For patients CNUH-11 and CNUH-51, we continued longitudinal monitoring of AFP abundance after recurrence, including changes observed following second-cycle treatment (Figure 4C). Patient CNUH-11, who initially achieved CR following surgical resection, exhibited reduced AFP abundance. Recurrence was identified 4.1 months after surgery, coinciding with an abrupt increase in cfDNA AFP levels. The patient subsequently underwent a second cycle of RFA, achieved CR again, and AFP abundance correspondingly decreased. Similarly, patient CNUH-51 initially demonstrated CR, with a decline in cfDNA AFP levels following the first TACE cycle. Recurrence was detected 8.9 months after treatment, accompanied by a sharp rise in AFP levels. The patient then received second-line treatment with RFA but experienced PD, and cfDNA AFP levels continued to spike at the subsequent follow-up visit. In parallel, we longitudinally monitored serum AFP protein levels and AFP^+^ CTC counts in these patients; however, no consistent trends were observed, likely due to the limited specificity of serum AFP and the low overall abundance of AFP^+^ CTCs (Figure S11). These findings suggest that longitudinal monitoring of AFP, specifically in cfDNA may provide a more reliable indicator for patient prognosis and recurrence monitoring compared to conventional serum AFP, highlighting the potential of cfDNA-based liquid biopsy as a superior tool for dynamic disease assessment in HCC.

## 4. Discussion

Serum AFP has been widely utilized in clinical practice for the management of HCC; however, its limited sensitivity and specificity, particularly in early-stage disease, significantly constrain its clinical utility. To overcome these limitations, we employed a bead-based capture assay to investigate AFP expression across alternative liquid biopsy biomarkers, including cfDNA and CTCs (Figure 1). These biomarkers demonstrated superior diagnostic performance, with cfDNA providing substantially improved diagnostic accuracy and CTCs offering high specificity for HCC detection (Figure 2). Moreover, dynamic changes in AFP levels derived from cfDNA and CTCs were significantly associated with treatment responses, whereas serum AFP levels exhibited limited predictive capability (Figure 3). In addition, changes in AFP abundance in cfDNA and CTCs following treatment were strongly associated with patients’ OS, further supporting their prognostic value (Figure 4). Although early post-treatment changes in AFP levels were not predictive of tumor recurrence—likely due to the temporal gap between sample collection and recurrence events—longitudinal monitoring of AFP levels in cfDNA enabled the detection of recurrence and facilitated the evaluation of treatment outcomes.

Our findings demonstrate that cfDNA-based AFP quantification outperforms conventional serum protein markers and CTCs in both diagnostic accuracy and prognostic value. This superior performance can be attributed to the biological characteristics of cfDNA: cancer patients typically exhibit significantly elevated cfDNA levels compared to healthy individuals, due to the high turnover rate of tumor cells, characterized by rapid proliferation, increased apoptosis, and necrosis, which release DNA fragments into the circulation [19,20]. For example, Wang et al. demonstrated significantly higher cfDNA concentrations in cancer patients than in healthy controls [19]. Importantly, cfDNA levels in cancer patients were also significantly higher than in patients with inflammatory conditions, indicating that cfDNA elevation is primarily driven by tumor development rather than by inflammation [20]. In contrast, tumor-associated proteins such as AFP, although secreted during tumor progression, can also be elevated in non-malignant inflammatory liver conditions, including hepatitis and cirrhosis, and are subject to metabolic clearance by the liver. This reduces their specificity and dynamic range as reliable biomarkers for HCC [21]. While our bead-based system captures total cfDNA from serum rather than exclusively tumor-derived fragments, these prior studies imply that a substantial fraction of cfDNA in cancer patients may have originated from tumor tissue and analysis of these DNA fragments can therefore provide clinically relevant information about tumor progression. By contrast, the lower sensitivity of CTCs is likely attributable to their limited abundance in peripheral blood and the technical challenges of detection using our system. Specifically, the modest capture efficiency of our bead-based platform (~70%) and reliance on EpCAM as a single capture marker may have led to underestimation of CTC counts, while defining AFP^+^ CTCs with a single biomarker based on IHC introduces additional technical limitations. Nevertheless, the high specificity of CTCs detected by our system reflects their direct derivation from malignant tissues, thereby providing genetic and phenotypic information that complements cfDNA-based analysis.

**Figure 5:**
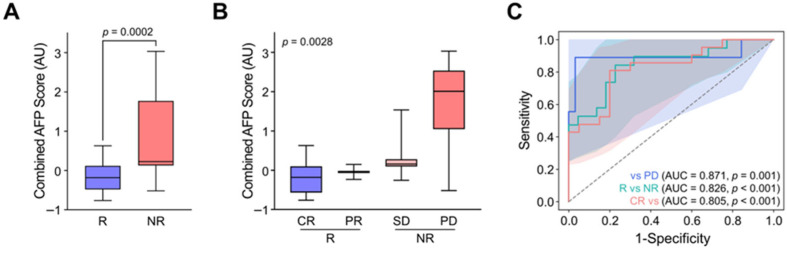
Enhanced treatment response stratification by integrating AFP levels from cfDNA and CTCs. (**A**) Combined AFP levels, calculated by integrating log changes in cfDNA-derived AFP abundance with post-treatment AFP^+^ CTC counts, significantly differentiated responders (CR/PR) from non-responders (SD/PD) (*p* = 0.0002). (**B**) The combined AFP levels enabled detailed stratification of therapeutic outcomes, with values increasing in accordance with response severity: CR, PR, SD, and PD. (**C**) ROC curve analysis demonstrated that the combined AFP index outperformed cfDNA and CTCs alone in predicting response categories, highlighting its potential utility for personalized response monitoring in HCC.

In this respect, we co-analyzed AFP levels in cfDNA and CTCs by merely adding the log changes in AFP abundance in cfDNA with post-treatment AFP^+^ CTC counts (combined AFP levels). Interestingly, the combined AFP level demonstrated significantly improved accuracy in discriminating patient responses. Non-responders (SD and PD) and responders (CR and PR) exhibited combined AFP levels of 0.23 ± 0.48 and −0.18 ± 0.27, respectively, with statistical significance (*p* = 0.0002) markedly higher than that achieved by cfDNA or CTCs alone (Figure 5A). Moreover, the combined AFP levels allowed for finer stratification of treatment responses, with values progressively increasing according to response category: −0.18 ± 0.27 for CR, −0.04 ± 0.19 for PR, 0.16 ± 0.05 for SD, and 2.01 ± 0.54 for PD (Figure 5B). Consequently, ROC analysis demonstrated that the combined AFP level not only outperformed cfDNA or CTCs alone in distinguishing responders from non-responders but also successfully classified specific response categories (Figure 5C). These findings highlight the potential of combined cfDNA and CTC-derived AFP analysis as a promising biomarker strategy, warranting further validation in prospective clinical trials for personalized monitoring and treatment stratification in HCC.

Although AFP levels derived from different tumor biomarkers demonstrated high accuracy in determining treatment responses, pre-treatment AFP levels did not correlate with patients’ pathological features. AFP levels obtained from serum proteins, cfDNA, and CTCs showed no strong association with tumor stage, multicentricity, tumor size, the presence of lymphovascular invasion, cirrhosis, or ascites, except for CTCs, which exhibited higher counts in patients with ascites (Figure S12). This discrepancy may arise since the baseline levels of cfDNA and CTCs can be influenced by multiple factors such as biological heterogeneity, tumor shedding dynamics, and clearance rates, which vary between patients. Supporting this, Sud et al. also reported that pre-treatment CTC counts did not correlate with metastatic burden in patients with oligometastatic disease, whereas dynamic changes in CTC levels more accurately reflected treatment outcomes [22]. Similarly, TERT promoter mutations detected in cfDNA were not indicative of tumor status (e.g., size or clinical stage) but were predictive of immunotherapy response and patient outcomes [23]. These findings suggest that although baseline biomarker levels may not reliably capture tumor burden, longitudinal monitoring of AFP dynamics in cfDNA and CTCs can provide more clinically meaningful information for patient management.

It should also be noted that although this study specifically focused on AFP expression in cfDNA and CTCs, the bead-based assay presented here has the potential to be extended for the analysis of other diagnostic and prognostic markers. For instance, methylation signatures or TERT promoter mutations in cfDNA have demonstrated strong diagnostic potential, while serum glypican-3 (GPC3) and protein induced by vitamin K absence or antagonist-II (PIVKA-II) have been associated with treatment response in HCC (Table S4) [24,25,26,27,28,29,30,31,32]. These markers have shown superior diagnostic and prognostic performance compared with conventional serum AFP analysis. Although our combined AFP analysis using CTCs and cfDNA demonstrated diagnostic and prognostic accuracy comparable to these biomarkers, we acknowledge that reliance on AFP alone remains an inherent limitation with respect to treatment personalization. Future studies should therefore extend this bead-based approach by incorporating analyses of tumor-specific mutations or methylation patterns within HCC-related genes in cfDNA, or by evaluating additional protein markers (e.g., GPC3) in CTCs alongside AFP. Such integration would enhance the clinical utility of this platform by enabling not only improved diagnosis and monitoring but also more precise applications in treatment selection and longitudinal patient management.

## 5. Conclusions

In this study, we demonstrated that AFP quantification from cfDNA and CTCs provides a more accurate and clinically informative assessment compared to conventional serum AFP levels for the diagnosis and prognosis of HCC. cfDNA-based AFP measurements exhibited superior diagnostic sensitivity, while CTC-derived AFP measurements provided high specificity. Importantly, dynamic changes in AFP levels from these liquid biopsy biomarkers were significantly associated with treatment responses and overall survival. Furthermore, the integration of cfDNA and CTC-derived AFP data improved response stratification beyond individual markers, offering a more comprehensive evaluation of patient outcomes. These findings highlight the potential of AFP analysis from CTCs and/or cfDNA as predictive tools in HCC management and suggest that serial monitoring of AFP expression changes in these biomarkers could be incorporated into clinical workflows to predict treatment efficacy at earlier stages. By validating these findings in larger clinical cohorts and incorporating additional biomarkers alongside AFP, this approach may further enhance the precision of HCC monitoring and facilitate its integration into personalized treatment strategies.

## Data Availability

Data are available upon reasonable request to the corresponding authors.

## Supplementary Materials

The following supporting information can be downloaded at https://www.mdpi.com/article/10.3390/bios15090579/s1, Table S1: Baseline demographic and clinical characteristics of study participants; Table S2: Summary of therapeutic strategies according to patient clinicopathological characteristics; Table S3: Summary of individual treatment responses and biomarker dynamics in HCC patients; Table S4: Comparison of biomarkers for diagnosis and prognosis of HCC; Figure S1: Representative IHC images of AFP^+^ CTCs isolated from peripheral blood of HCC patients; Figure S2: Photographic images of bare alginate beads, PDA/SiO_2_-coated beads, and aEpCAM-coated beads; Figure S3: Decision tree summarizing the overall workflow of this study, from sample collection to bead-based analysis of AFP levels in CTCs and cfDNA; Figure S4: Confirmation of aEpCAM conjugation onto alginate beads; Figure S5: Capture sensitivity of the Hep3B HCC cell line using aEpCAM-coated beads; Figure S6: Fluorescence analysis of PDA/SiO_2_-coated beads compared with bare beads after incubation with fluorescently labeled nucleic acids; Figure S7: Diagnostic performance of each biomarker at the optimal Youden index; Figure S8: Expression profile of liver function biomarkers between non-cancerous individuals and HCC patients; Figure S9: ROC analysis of conventional liver function markers for distinguishing HCC patients from healthy individuals; Figure S10: ROC analysis of dynamic changes in conventional liver function markers for distinguishing treatment non-responders from responders; Figure S11: Longitudinal monitoring of serum AFP levels and AFP^+^ CTC counts in relation to recurrence and second-line treatment; Figure S12: Pre-treatment AFP levels in serum, cfDNA, and CTCs according to patient clinicopathological characteristics.

## Abbreviations

The following abbreviations are used in this manuscript:

AFP	Alpha-fetoprotein
cfDNA	Cell-free DNA
CTC	Circulating tumor cell
qPCR	Quantitative polymerase chain reaction
IHC	Immunohistochemistry
PBMC	Peripheral blood mononuclear cell
PDA/SiO_2_	Polydopamine–silica hybrid
aEpCAM	Anti-epithelial cell adhesion molecule
GAPDH	Glyceraldehyde-3-phosphate dehydrogenase
ECLIA	Electrochemiluminescence immunoassay
CR	Complete response
PR	Partial response
SD	Stable disease
PD	Progressive disease
OS	Overall survival
RFS	Recurrence-free survival
TACE	Transarterial chemoembolization
RFA	Radiofrequency ablation
KM	Kaplan–Meier
ctDNA	Circulating tumor DNA
