# Supplementary material for: Cell-Free DNA Versus Circulating Tumor Cells: A Pilot Study of Alpha-Fetoprotein Analysis for Diagnosis and Treatment Monitoring in Hepatocellular Carcinoma
Source: Biosensors (Basel). 2025 Sep 4;15(9):579. doi: 10.3390/bios15090579 (PMC12467720; doi:10.3390/bios15090579)
Supplement: Supplementary file 1 [file biosensors-15-00579-s001.zip › biosensors-3784167-supplementary.pdf]

Supplementary Information

Cell-free DNA versus Circulating Tumor Cells: A Pilot Study of Alpha-Fetoprotein Analysis for Diagnosis and Treatment Monitoring in Hepatocellular Carcinoma

Ga Young Moon[1,a], Hyun Sung Park[1,a], Ha Neul Kim[2 3,a], Hei-Gwon Choi[2,3], Yonghan Han[1], Hyuk Soo Eun[2,3,4,] , Tae Hee Lee[5,*] , Jiyoon Bu[1,6,7,8,*]*

[1] Department of Biological Sciences and Bioengineering, Inha University, 100 Inha-ro, Michuhol-gu, Incheon, 22212, Republic of Korea; gayeong056@inha.edu (G.Y.M.); hyunseong@inha.edu (H.S.P.); choko9219@inha.edu (Y.H.)

[2] Department of Medical Science, Chungnam National University, 266 Munhwa-ro, Jung-gu, Daejeon 35015, Republic of Korea; tsb04254@o.cnu.ac.kr (H.N.K.); hundred4120@cnu.ac.kr (H.-G.C.)

[3] Department of Internal Medicine, College of Medicine, Chungnam National University 266 Munhwa-ro, Jung-gu, Daejeon 35015, Republic of Korea

[4] Department of Internal Medicine, Chungnam National University Hospital, 282 Munwha-ro, Jung-gu, Daejeon 35015, Republic of Korea

[5] Department of Biomedical Laboratory Science, Daegu Health College, Chang-ui building, 15 Yeongsong-ro, Buk-gu, Daegu, 41453, Republic of Korea

[6] Biomedical Research Institute, Inha University Hospital, 27 Inhang-ro, Jung-gu, Incheon 22332, Republic of Korea

[7] Department of Biological Engineering, Inha University, 100 Inha-ro, Michuhol-gu, Incheon 22212, Republic of Korea

[8] Biohybrid Systems Research Center, Inha University, 100 Inha-ro, Michuhol-gu, Incheon 22212, Republic of Korea

*Correspondence: hyuksoo@cnuh.co.kr (H.S.E.); taehee1155@gmail.com (T.H.L.); jbu@inha.ac.kr (J.B.)
[a] These authors contributed equally to this work.

Table S1. Baseline demographic and clinical characteristics of study participants. Note that the numbers are given as median (range).

Cohorts	Sex	Age (years)	Height (cm)	Weight (kg)	cfDNA AFP Level (log $2^{-\Delta t}$)	AFP^+ CTCs (cells/mL)*	Serum AFP (ng/mL)	Other Serum Markers			
								AST (IU/L)	ALT (IU/L)	ALP (IU/L)	Albumin (g/dL)
HD	Female: 4<br>Male: 6<br>(Total: 10)	37<br>(25-44)	171<br>(160-187)	70<br>(52-94)	−0.337<br>(−0.739-0.021)	0<br>(0 – 0)	5.06<br>(1.98 -24.8)	24<br>(16-213)	26<br>(12-53)	30.5<br>(10-62)	4.65<br>(4-5.2)
HCC	Female: 6<br>Male: 41<br>(Total: 47)	63<br>(42-88)	165<br>(146-177)	68<br>(49-91)	0.486<br>(0.018-0.832)	0.357<br>(0 - 2)	6.28<br>(1.11 – 2,765)	34<br>(17-887)	23<br>(9-588)	87.5<br>(29-262)	4.1<br>(2.7-4.8)

* AFP^+ CTC counts are expressed in average (range).

Table S2. Summary of therapeutic strategies according to patient clinicopathological characteristics. Patients with HCC underwent different treatment modalities based on individualized clinical judgment, including radiofrequency ablation (RFA), transarterial chemoembolization (TACE), surgical resection, or radiotherapy (RT). Treatment decisions were guided by clinical parameters such as tumor size, number, stage, and liver function status.

		**Surgical resection**	**Surgical resection + RFA**	**RFA**	**TACE**	**Others (Radiotherapy or LT)**
UICC Stage	I	3	3	7	2	0
	II	7	4	4	7	0
	III	0	0	0	1	0
	IV	1	0	1	0	2
Multicentricity	Y	2	1	1	5	1
	N	9	6	11	5	1
Vascular Invasion	Y	0	0	1	0	2
	N	11	7	11	10	0
Cirrhosis	Y	7	3	10	7	2
	N	4	4	2	3	0
Ascties	Y	0	0	2	0	1
	N	11	7	10	10	1
Tumor Size (Median)	>2 cm	7 (3.2)	4 (3.4)	4 (3.9)	6 (5.1)	2 (11.0)
	<=2 cm	4 (1.1)	3 (1.2)	8 (1.3)	4 (1.4)	0

Table S3. Summary of individual treatment responses and biomarker dynamics in HCC patients. For each case, pre- and post-treatment AFP levels derived from CTCs, cfDNA, and serum are provided to evaluate the predictive performance of each biomarker.

Patient ID	Treatment	Recist1.1	Serum AFP (ng/mL)		cfDNA AFP (log $2^{-\Delta t}$)		AFP^{+} CTCs (cells/mL)	
			pre	post	pre	post	pre	post
CNUH-36	RFA	CR	1.11	1.31	0.573	0.581	1	2
CNUH-42	RFA	CR	4.60	NA	0.531	0.995	1	1
CNUH-40	RFA	CR	3.50	3.91	0.348	0.882	2	1
CNUH-43	RFA	CR	6.73	5.51	0.370	0.594	0	0
CNUH-33	RFA	PD	670	NA	0.566	NA	0	0
CNUH-38	RFA	PD	1.85	1.7	0.257	0.483	0	0
CNUH-32	RFA	PD	8.67	NA	0.773	NA	0	0
CNUH-35	RFA	SD	23.1	3.26	0.444	0.616	0	0
CNUH-37	RFA	SD	3.57	2.13	0.170	0.238	0	0
CNUH-41	RFA	SD	10.3	38.1	0.394	-0.314	0	0
CNUH-39	RFA	SD	2.30	2.32	0.458	0.502	0	1
CNUH-34	RFA	SD	2500	3.08	0.572	-0.194	0	0
CNUH-51	TACE	CR	183	5.73	0.570	0.320	1	0
CNUH-49	TACE	CR	2765	2765	0.606	0.745	0	0
CNUH-47	TACE	CR	14.3	12.5	0.400	0.167	0	0
CNUH-46	TACE	PR	2.98	2.17	0.177	0.200	0	0
CNUH-48	TACE	PR	7.06	3.52	0.019	0.461	0	0
CNUH-50	TACE	SD	2.73	2.45	0.624	0.743	0	0
CNUH-45	TACE	SD	14.9	11.5	0.401	0.840	0	0
CNUH-44	TACE	SD	125	4.28	0.465	0.875	0	0
CNUH-53	TACE	SD	2.46	4.61	0.334	0.481	0	0
CNUH-52	TACE	SD	9.36	9.44	0.430	0.597	0	0
CNUH-25	Resection + RFA	CR	37.5	37.5	0.526	0.634	0	0
CNUH-31	Resection + RFA	CR	11.0	9.36	0.589	0.335	0	0
CNUH-26	Resection + RFA	CR	3.9	4.04	0.357	-0.291	0	0
CNUH-29	Resection + RFA	CR	3.22	2.73	0.751	0.601	1	0
CNUH-28	Resection + RFA	CR	7.27	5.75	0.669	0.15	1	0
CNUH-30	Resection + RFA	PD	1.54	NA	0.657	NA	2	0
CNUH-27	Resection + RFA	SD	4.52	4.45	0.653	0.214	0	0
CNUH-11	Resection	CR	2.91	2.56	0.359	0.134	2	0
CNUH-15	Resection	CR	3.07	3.49	0.556	-0.124	0	0
CNUH-21	Resection	CR	2.96	3.27	0.425	-0.188	0	0
CNUH-20	Resection	CR	2.58	NA	0.329	0.352	1	2
CNUH-12	Resection	CR	11.5	10.4	0.535	0.419	0	0
CNUH-18	Resection	CR	5.74	2.81	0.606	0.756	0	0
CNUH-17	Resection	CR	4.16	5.81	0.506	0.292	0	0
CNUH-14	Resection	CR	96.9	2.69	0.58	0.553	0	0
CNUH-16	Resection	PD	5.83	3.67	0.097	0.724	0	0
CNUH-22	Resection	PD	69.9	4.88	0.832	0.687	1	0
CNUH-13	Resection	PR	4.77	3.51	0.163	-0.258	0	0
CNUH-54	Others	PD	28.5	NA	0.436	0.973	2	2
CNUH-55	Others	PD	63.8	146	0.572	0.605	0	3

Table S4. Comparison of biomarkers for diagnosis and prognosis of HCC.

Biomarkers	Target biomarker	Patients	Diagnostic/Prognostic Performance	Treatment	Reference
cfDNA methylation panel	cg04645914, cg06215569, cg23663760, cg13781744, and cg07610777	cfDNA methylation HCC (n=22), non-HCC (n=22)	Demonstrated AUC-ROC = 0.953 for HCC diagnosis.	NA	[24]
cfDNA methylation panel	cg25026480, cg14774440, cg18054281 and cg00638020	cfDNA hypomethylation HCC (n=22), non-HCC (n=22)	Demonstrated AUC-ROC = 0.971 for HCC diagnosis.	NA	[24]
cfDNA methylation panel	5hmC	HCC (n=103), non-HCC (n=167)	Demonstrated AUC-ROC = 0.939 for HCC diagnosis.	NA	[25]
cfDNA methylation panel	Septin9, GRASP, TSPYL5 and SPINT2	HCC (n=205), non-HCC (n=308)	Demonstrated AUC-ROC = 0.940 for HCC diagnosis.	NA	[26]
cfDNA methylation panel	cg23461741, cg06482904, cg25574765, cg07459019, cg20490031, cg01643250, cg11397370 and cg11825899	HCC (n=383), non-HCC (n=275)	Demonstrated AUC-ROC = 0.944 for HCC diagnosis. Low-risk group (cp-score < -0.24) has higher OS than high-risk group (cp-score > -0.24) (p = 0.0014). Signficant difference in cp-socre among responders vs. non-responders (p < 0.0001).	Chemotherapy, Surgical resection	[27]
TERT promoter mutations	TERT promoter	TERT mutation positive patients (n=71) and TERT mutaion negative patients (n=64) are HCC patients (n=130)	Median OS: 12.8 months (mutation) vs. 27.0 months (non-mutation), p < 0.001.	Systemic chemotherapy (Sorafenib or Lenvatinib) or TACE	[28]
GPC3	GPC 3	AFP negative HCC (n=104)	Demonstrated AUC-ROC = 0.751 for the detection of AFP-positive HCC patients. Median OS: 42.5 months (GP3-low) vs. 28.2 months (GPC3-high), p = 0.0018.	NA	[29]
PIVKA-II	PIVKA-II	AFP negative HCC (n=104)	Demonstrated AUC-ROC = 0.925 for the detection of AFP-positive HCC patients. Median OS: 40.0 months (PIVKA-II-low) vs. 30.1 months (PIVKA-II-high), p = 0.0015.	NA	[29]
PIVKA-II	PIVKA-II	HCC (n=145), HCs (n=101)	Mean PFS: 448 months (PIVKA-II-negative) vs. 163 months (PIVKA-II-positive), p < 0.05.	Surgical resection	[30]
PIVKA-II	PIVKA-II	HCC (n=61)	Significant diffence in PIVKA-II levels between ICI responder vs. non-responder (p < 0.001).	ICI, MKI, Chemothrapy	[31]
GPC3	GPC3	HCC (n=25), recurrence (n=14), non recurrence (n=11)	Median PFS: 544 days (GP3-positive) vs. not recurred (GPC3-negative) upon surgery.	Surgical resection	[32]

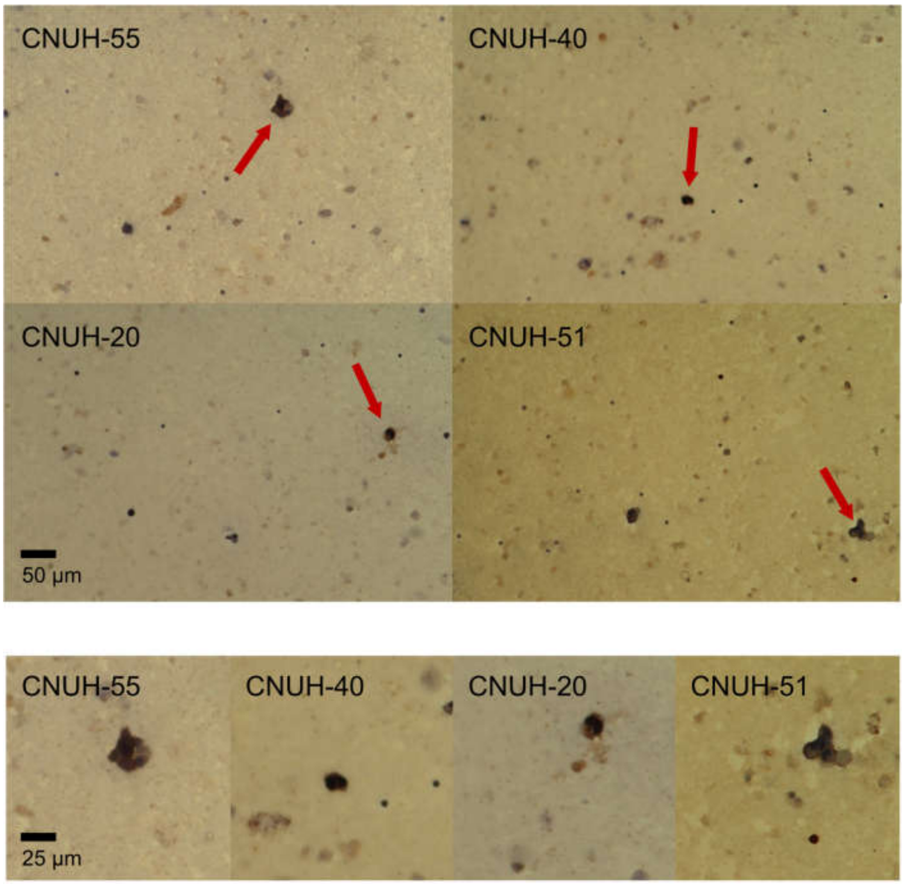

Figure S1. Representative IHC images of AFP^{+} CTCs isolated from peripheral blood of HCC patients. AFP^{+} CTCs were identified by brownish cytoplasmic staining for AFP, with morphological features including a cell diameter >12 μm and a higher nuclear-to-cytoplasmic ratio compared to background leukocytes. Red arrows indicate AFP-positive cells.

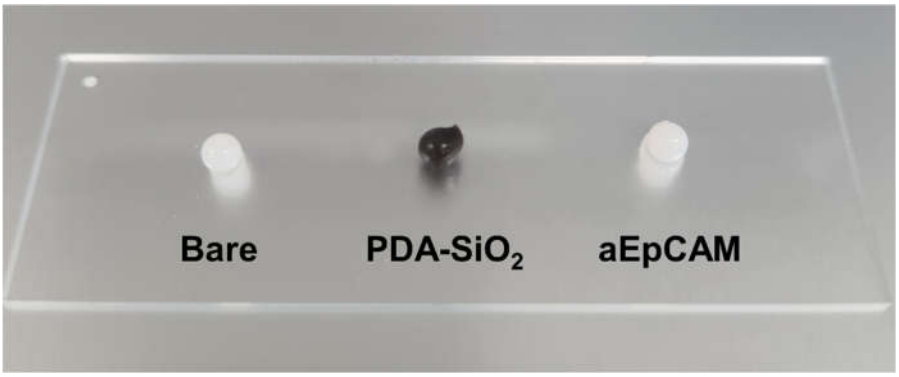

Figure S2. Photographic images of bare alginate beads, PDA/SiO_2-coated beads, and aEpCAM-coated beads.

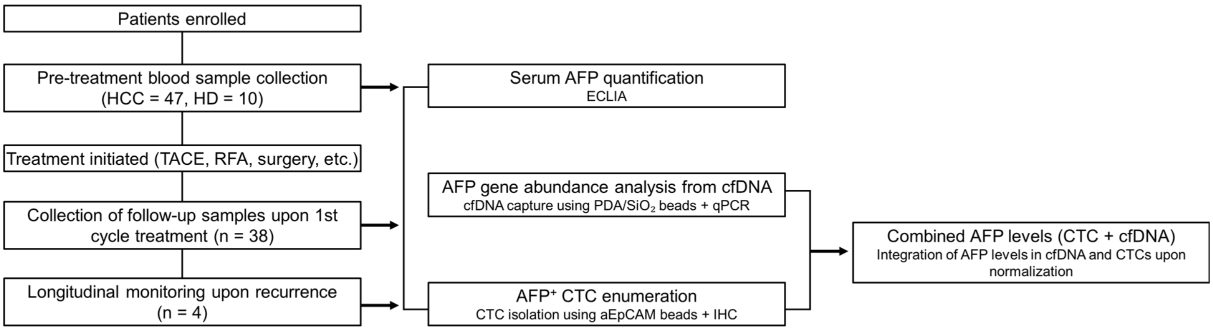

Figure S3. Decision tree summarizing the overall workflow of this study, from sample collection to bead-based analysis of AFP levels in CTCs and cfDNA.

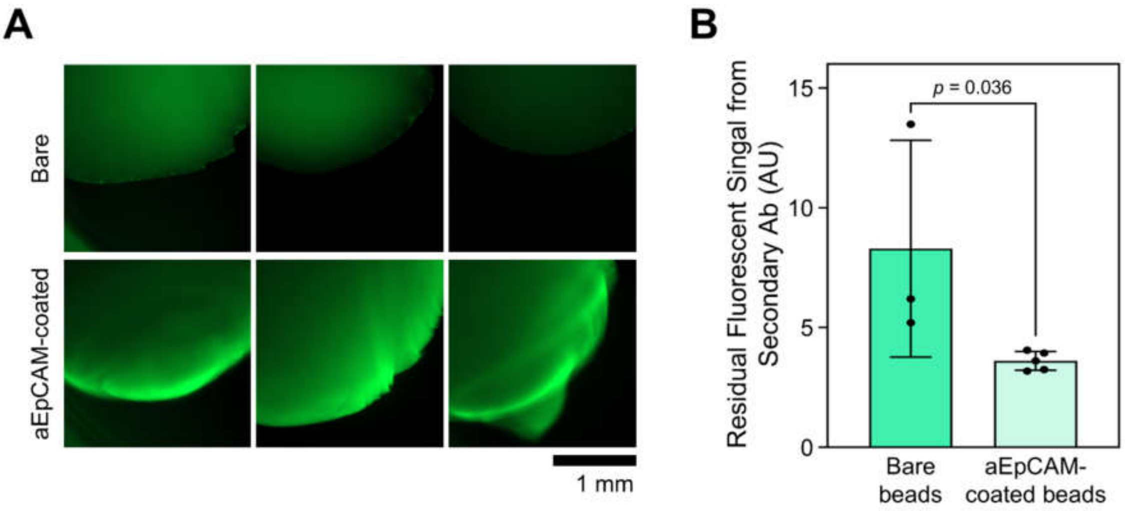

Figure S4. Confirmation of aEpCAM conjugation onto alginate beads. (**A**) Representative fluorescence microscopy images of bare and aEpCAM-functionalized alginate beads following incubation with Alexa Fluor 488-labeled secondary antibodies. (**B**) Quantification of residual fluorescence intensity, showing significantly reduced signal in aEpCAM-coated beads compared with bare beads, confirming successful antibody conjugation.

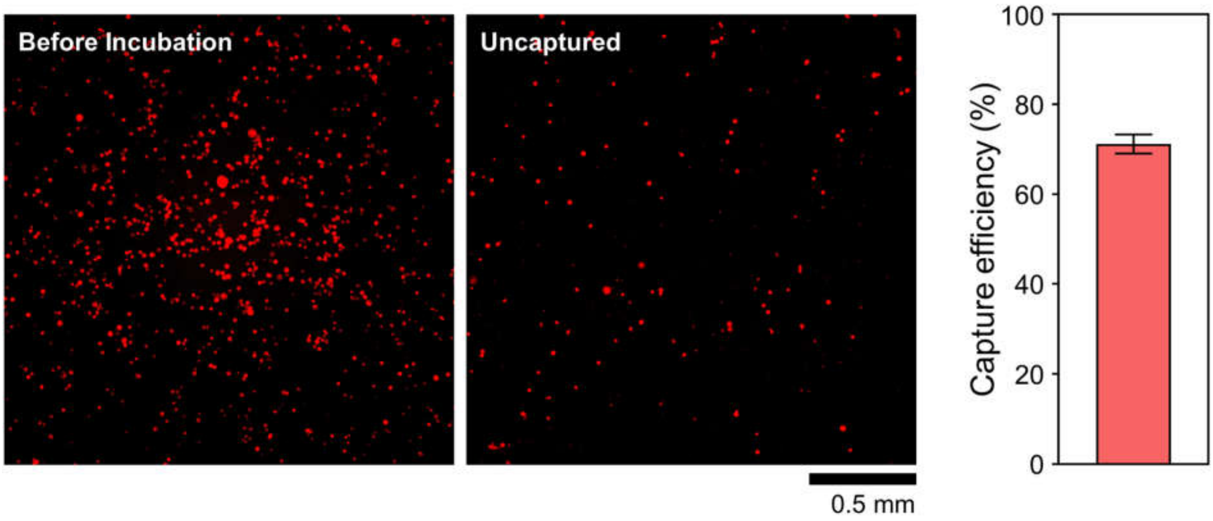

Figure S5. Capture sensitivity of the Hep3B HCC cell line using aEpCAM-coated beads.

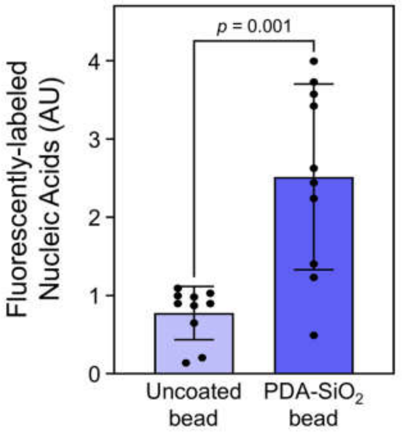

Figure S6. Fluorescence analysis of PDA/SiO_2-coated beads compared with bare beads after incubation with fluorescently labeled nucleic acids.

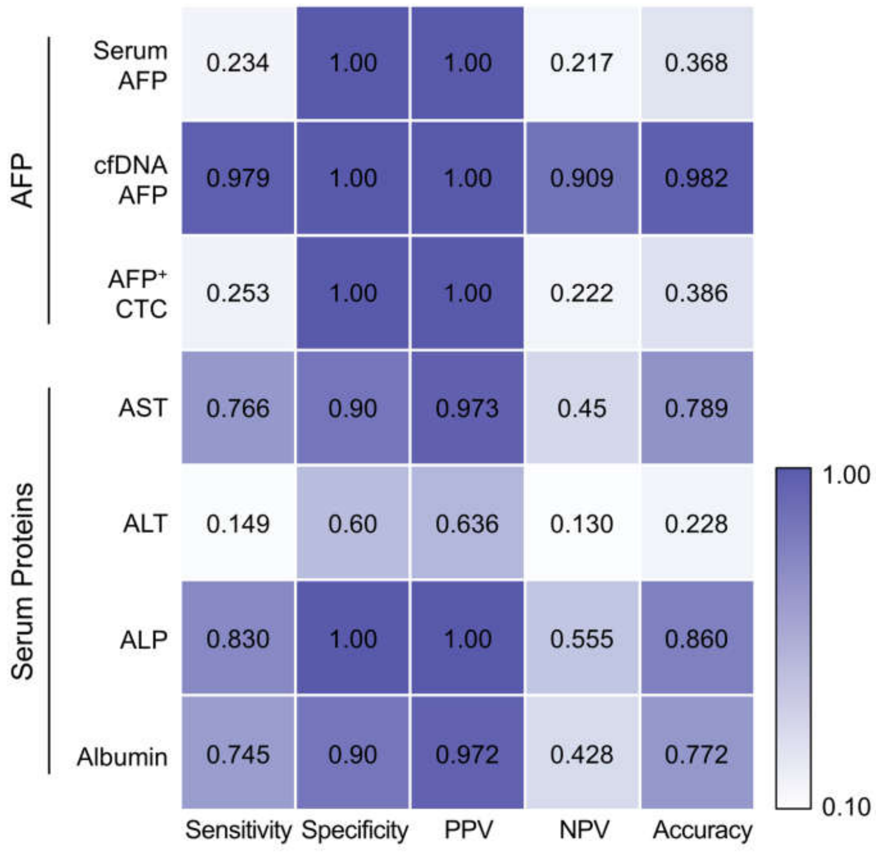

Figure S7. Diagnostic performance of each biomarker at the optimal Youden index. Summary of diagnostic metrics including sensitivity, specificity, positive predictive value (PPV), negative predictive value (NPV), and overall accuracy for each biomarker—serum AFP, cfDNA AFP abundance, AFP^+ CTCs, and conventional liver function tests (AST, ALT, ALP, and albumin)—calculated at the threshold corresponding to the maximum Youden index.

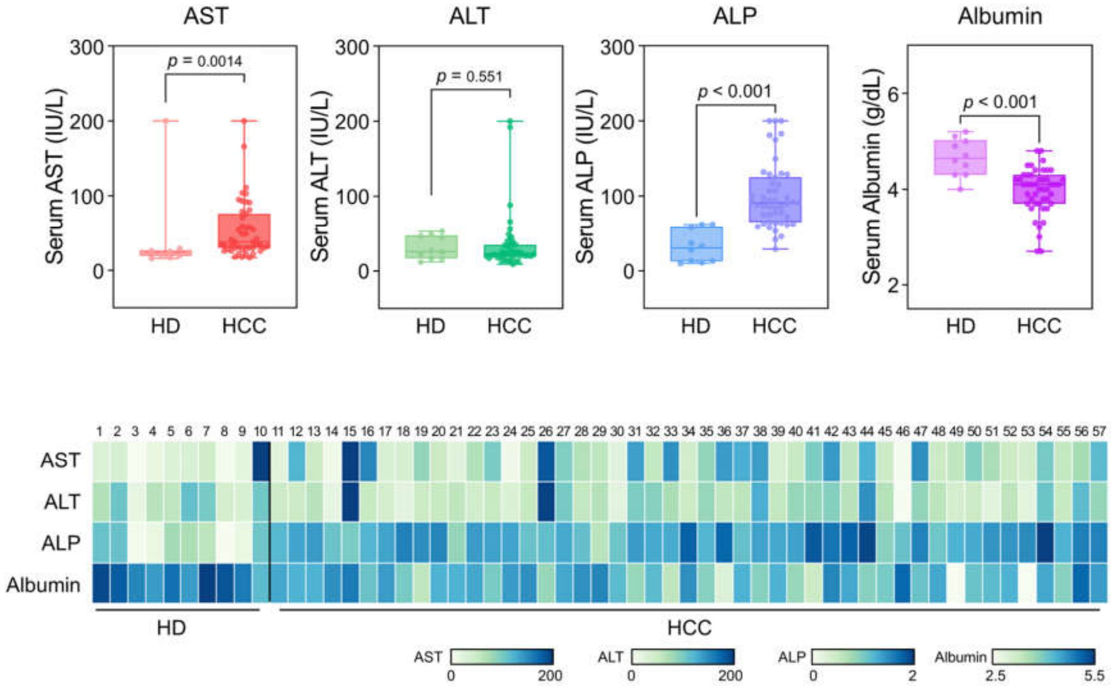

Figure S8. Expression profile of liver function biomarkers between non-cancerous individuals and HCC patients. Box plots and heatmap illustrating serum levels of conventional liver function markers, including AST, ALT, ALP, and albumin, in healthy donors (n = 10) and patients with HCC (n = 47).

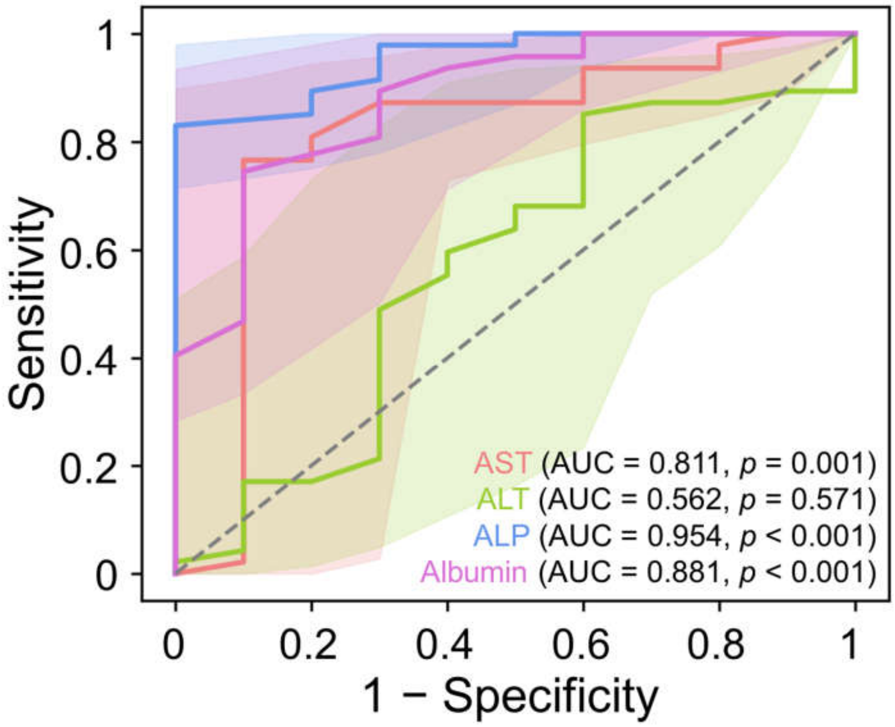

Figure S9. ROC analysis of conventional liver function markers for distinguishing HCC patients from healthy individuals.

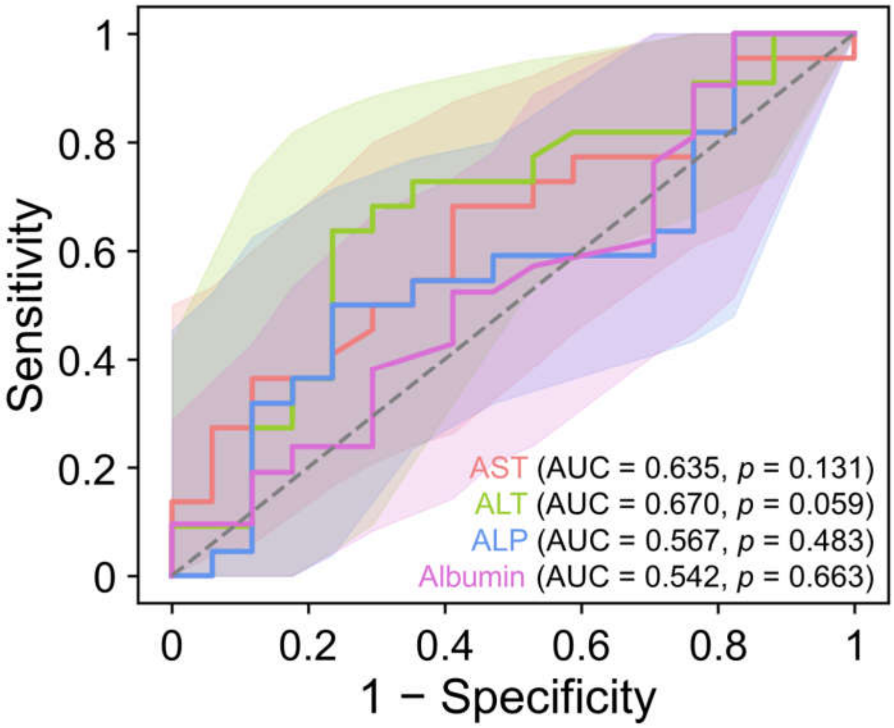

Figure S10. ROC analysis of dynamic changes in conventional liver function markers for distinguishing treatment non-responders from responders.

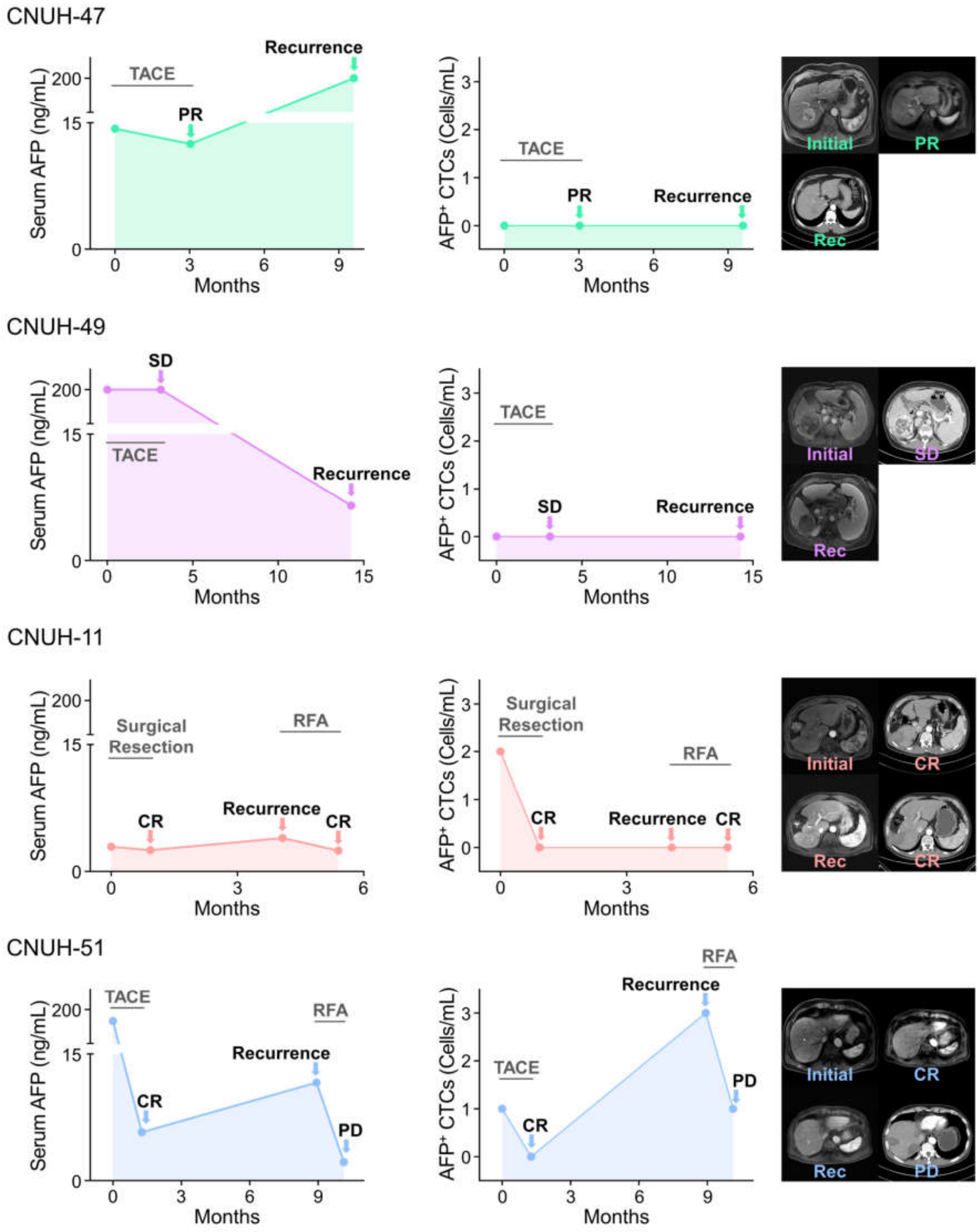

Figure S11. Longitudinal monitoring of serum AFP levels and AFP^{+} CTC counts in relation to recurrence and second-line treatment.

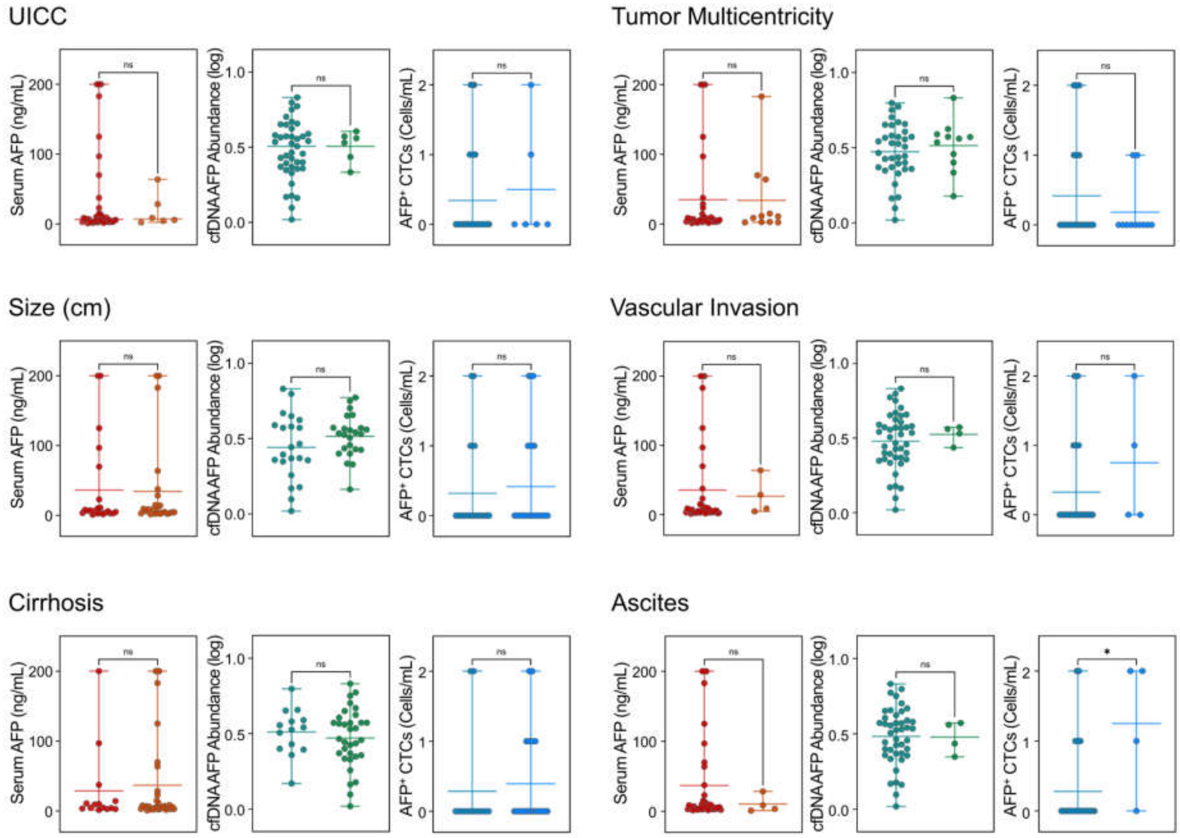

Figure S12. Pre-treatment AFP levels in serum, cfDNA, and CTCs according to patient clinicopathological characteristics.

References

24. Hlady, R.A.; Zhao, X.; Pan, X.; Yang, J.D.; Ahmed, F.; Antwi, S.O.; Giama, N.H.; Patel, T.; Roberts, L.R.; Liu, C.; Robertson, K.D. Genome-wide discovery and validation of diagnostic DNA methylation-based biomarkers for hepatocellular cancer detection in circulating cell free DNA. Theranostics **2019**, 9, 7239-7250, doi:10.7150/thno.35573.

25. Cai, Z.; Zhang, J.; He, Y.; Xia, L.; Dong, X.; Chen, G.; Zhou, Y.; Hu, X.; Zhong, S.; Wang, Y.; Chen, H.; Xie, D.; Liu, X.; Liu, J. Liquid biopsy by combining 5-hydroxymethylcytosine signatures of plasma cell-free DNA and protein biomarkers for diagnosis and prognosis of hepatocellular carcinoma. ESMO Open **2021**, 6, 100021, doi:https://doi.org/10.1016/j.esmoop.2020.100021.

26. Guo, D.Z.; Huang, A.; Wang, Y.C.; Zhou, S.; Wang, H.; Xing, X.L.; Zhang, S.Y.; Cheng, J.W.; Xie, K.H.; Yang, Q.C.; Ma, C.C.; Li, Q.; Chen, Y.; Su, Z.X.; Fan, J.; Liu, R.; Liu, X.L.; Zhou, J.; Yang, X.R. Early detection and prognosis evaluation for hepatocellular carcinoma by circulating tumour DNA methylation: A multicentre cohort study. Clin Transl Med **2024**, 14, e1652, doi:10.1002/ctm2.1652.

27. Xu, R.H.; Wei, W.; Krawczyk, M.; Wang, W.; Luo, H.; Flagg, K.; Yi, S.; Shi, W.; Quan, Q.; Li, K.; Zheng, L.; Zhang, H.; Caughey, B.A.; Zhao, Q.; Hou, J.; Zhang, R.; Xu, Y.; Cai, H.; Li, G.; Hou, R.; Zhong, Z.; Lin, D.; Fu, X.; Zhu, J.; Duan, Y.; Yu, M.; Ying, B.; Zhang, W.; Wang, J.; Zhang, E.; Zhang, C.; Li, O.; Guo, R.; Carter, H.; Zhu, J.K.; Hao, X.; Zhang, K. Circulating tumour DNA methylation markers for diagnosis and prognosis of hepatocellular carcinoma. Nat Mater **2017**, 16, 1155-1161, doi:10.1038/nmat4997.

28. Hirai, M.; Kinugasa, H.; Nouso, K.; Yamamoto, S.; Terasawa, H.; Onishi, Y.; Oyama, A.; Adachi, T.; Wada, N.; Sakata, M.; Yasunaka, T.; Onishi, H.; Shiraha, H.; Takaki, A.; Okada, H. Prediction of the prognosis of advanced hepatocellular carcinoma by TERT promoter mutations in circulating tumor DNA. J Gastroenterol Hepatol **2021**, 36, 1118-1125, doi:10.1111/jgh.15227.

29. Lin, Y.; Ma, Y.; Chen, Y.; Huang, Y.; Lin, J.; Xiao, Z.; Cui, Z. Diagnostic and prognostic performance of serum GPC3 and PIVKA-II in AFP-negative hepatocellular carcinoma and establishment of nomogram prediction models. BMC Cancer **2025**, 25, 721, doi:10.1186/s12885-025-14025-y.

30. Tian, S.; Chen, Y.; Zhang, Y.; Xu, X. Clinical value of serum AFP and PIVKA-II for diagnosis, treatment and prognosis of hepatocellular carcinoma. J Clin Lab Anal **2023**, 37, e24823, doi:10.1002/jcla.24823.

31. Chen, S.C.; Ho, H.L.; Liu, C.A.; Hung, Y.P.; Chiang, N.J.; Chen, M.H.; Chao, Y.; Yang, M.H. PIVKA-II as a surrogate biomarker for therapeutic response in Non-AFP-secreting hepatocellular carcinoma. BMC Cancer **2025**, 25, 199, doi:10.1186/s12885-025-13568-4.

32. Ofuji, K.; Saito, K.; Suzuki, S.; Shimomura, M.; Shirakawa, H.; Nobuoka, D.; Sawada, Y.; Yoshimura, M.; Tsuchiya, N.; Takahashi, M.; Yoshikawa, T.; Tada, Y.; Konishi, M.; Takahashi, S.; Gotohda, N.; Nakamoto, Y.; Nakatsura, T. Perioperative plasma glypican-3 level may enable prediction of the risk of recurrence after surgery in patients with stage I hepatocellular carcinoma. Oncotarget **2016**, 8.
